# CD4^+^CD8^+^ T-Lymphocytes in Xenogeneic and Human Graft-versus-Host Disease

**DOI:** 10.3389/fimmu.2020.579776

**Published:** 2020-11-24

**Authors:** Kutaiba Alhaj Hussen, David Michonneau, Vincent Biajoux, Seydou Keita, Laetitia Dubouchet, Elisabeth Nelson, Niclas Setterblad, Helene Le Buanec, Jean-David Bouaziz, Fabien Guimiot, Gérard Socié, Bruno Canque

**Affiliations:** ^1^INSERM U976, Université de Paris, École Pratique des Hautes Études/PSL Research University, Institut de Recherche Saint Louis, Paris, France; ^2^Service d’Hématologie Biologique, Hôpital Tenon, Hôpitaux Universitaires de l’Est Parisien, Assistance Publique Hôpitaux de Paris, Paris, France; ^3^INSERM U976, Université de Paris; Service d’hématologie-greffe, AP-HP, Hôpital Saint-Louis, Institut de Recherche Saint Louis, Paris, France; ^4^Plateforme d’Imagerie et de Tri Cellulaire, Institut de Recherche Saint Louis, Paris, France; ^5^INSERM U976, Dermatology Department, Hôpital Saint-Louis, Institut de Recherche Saint Louis, Paris, France; ^6^INSERM UMR 1141, Service de Biologie du Développement, Université de Paris, Hôpital Robert-Debré, AP-HP, Paris, France

**Keywords:** allogeneic hematopoietic stem cell transplantation, graft-versus-host disease, xenograft mouse model, CD4^+^CD8^+^ T lymphocytes, immunoregulatory diversion

## Abstract

Mechanisms driving acute graft-versus-host disease (aGVHD) onset in patients undergoing allogeneic hematopoietic stem cell transplantation (allo-HSCT) are still poorly understood. To provide a detailed characterization of tissue-infiltrating T lymphocytes (TL) and search for eventual site-specific specificities, we developed a xenogeneic model of aGVHD in immunodeficient mice. Phenotypic characterization of xenoreactive T lymphocytes (TL) in diseased mice disclosed a massive infiltration of GVHD target organs by an original CD4^+^CD8^+^ TL subset. Immunophenotypic and transcriptional profiling shows that CD4^+^CD8^+^ TL comprise a major PD1^+^CD62L^−/+^ transitional memory subset (>60%) characterized by low level expression of cytotoxicity-related transcripts. CD4^+^CD8^+^ TL produce high IL-10 and IL-13 levels, and low IL-2 and IFN-γ, suggestive of regulatory function. In vivo tracking of genetically labeled CD4^+^ or CD8^+^ TL subsequently found that CD4^+^CD8^+^ TL mainly originate from chronically activated cytotoxic TL (CTL). On the other hand, phenotypic profiling of CD3^+^ TL from blood, duodenum or rectal mucosa in a cohort of allo-HSCT patients failed to disclose abnormal expansion of CD4^+^CD8^+^ TL independent of aGVHD development. Collectively, our results show that acquisition of surface CD4 by xenoreactive CD8^+^ CTL is associated with functional diversion toward a regulatory phenotype, but rule out a central role of this subset in the pathogenesis of aGVHD in allo-HSCT patients.

## Introduction

Allogeneic hematopoietic stem-cell transplantation (HSCT) is an effective therapy for hematological malignancies. While hematopoietic stem cells (HSC) ensure hematopoietic recovery, donor T lymphocytes (TL) display a dual activity. Although capable of destroying residual tumor cells through the Graft-Versus Leukemia reaction (GVL) they also frequently cause acute Graft-Versus-Host Disease (aGVHD). With incidence still lying between 40% and over 80% (1, 2), aGVHD remains the main factor contributing to non-relapse morbidity in transplanted patients (3). The mechanisms driving aGVHD onset and dissemination are still poorly understood. Besides genetic disparity between donor and recipient, there is evidence that pro-inflammatory signals emanating from host tissues or organs injured by pre-transplants conditioning also contribute to disease propagation (4). In parallel, activation of donor and recipient antigen-presenting cells, as well as increased expression of adhesion and costimulatory molecules and high chemokine production levels (5), exacerbate donor T cells activation resulting in accelerated blood extravasation followed by *in situ* amplification within aGVHD target organs (6, 7). Alloreactive TL mediate tissue destruction either directly through granzyme/perforin-dependent cytolytic activity or Fas/FasL interactions (8, 9), or indirectly through the recruitment or activation of monocyte/macrophages and neutrophils.

The role played by CD4^+^ helper and CD8^+^ cytotoxic TL in the pathophysiology of aGVHD has also been extensively documented in the mouse (7, 10). Despite evidence that development of a full-blown disease depends on cooperative interactions between helper and cytotoxic TL (11–15), the specific role played by each of these subsets has not been fully elucidated. Although earlier reports in the mouse have shown that memory TL are less efficient than their naïve counterparts in inducing aGVHD (16, 17), there is no clear evidence that this applies in the human setting (18). Similarly, current knowledge does not support the idea that human aGVHD might correspond solely to a T_H_1-, T_H_2- (19, 20) or Th17-mediated disease (21, 22), and although regulatory T (23, 24) or NKT cells (25) can prevent aGVHD in the mouse whether they contribute to limit disease incidence or severity in the clinic remains unclear (26, 27).

Over the past years there has also been a growing interest in the biology of CD4^+^CD8^+^ double positive (DP) TL. Since their identification some 40 years ago (28), DP T cells have been detected at a higher frequency in the blood and tissues of patients with viral infections (CMV, EBV, HIV, HCV), cancer (breast, colorectal, urologic, melanoma), hematological disorders (cutaneous T cell lymphoma, aplastic anemia) or autoimmune (rheumatoid arthritis, atopic dermatitis, systemic sclerosis) disorders, and inflammatory bowel or Chagas disease (29). Whether DP TL differentiate from single positive CD4^+^ helper or CD8^+^ cytotoxic TL or if they vary in both origin and function in a context-dependent manner is still incompletely resolved (30, 31). Inasmuch as diverse CD4^hi^CD8^dim^ (32, 33), CD4^hi^CD8^hi^ (34), or CD4^dim^CD8^hi^ (35), TL subsets with cytotoxic or immunoregulatory properties have been described, an emerging concept is that whereas CD4^+^CD8α^+^β^-^ TL should differentiate from CD4^+^ helper TL, their CD4^+^CD8α^+^β^+^ counterparts originate primarily from conventional cytotoxic TL. While most current evidence suggests that ThPOK downmodulation in chronically activated CD4^+^ helper T cells is associated with upregulation of a cytotoxic differentiation program (36), mechanisms driving reciprocal acquisition of surface CD4 by cytotoxic TL are still poorly documented (32, 37).

Here, using a xenogeneic model of aGVHD we demonstrate that persistent activation of tissue-infiltrating CD8^+^ CTL promotes CD4 upregulation associated with functional diversion toward a regulatory phenotype. Analysis of a series of allo-HSCT patients failed to provide evidence that DP TL could play a central role in the pathogenesis of aGVHD in the clinic suggesting that despite massive polyclonal amplification the mouse environment does not allow for optimal activation/maturation of human TL.

## Materials and Methods

### Human Sample Collection

Cytapheresis from adult donors were provided by the Etablissement Français du Sang Unit of Hôpital Saint-Louis (Paris). Blood mononuclear cells separated by Ficoll-Hypaque centrifugation (Pancoll, PAN Biotech GmbH) were frozen in heat-inactivated fetal calf serum (FCS) supplemented with 10% DMSO and stored in liquid nitrogen until use. All patients from Saint Louis hospital signed consent for blood, plasma and stools collection, including consent for genetic study (MICROBIOTE-T3 collection, IRB 00003835, CNIL 104706). All patients had a blood sample at day 90 post-transplant in the absence of GVHD or before administration of immunosuppressive treatment for newly-diagnosed acute GVHD. All patients signed consent for registration and use of clinical and biological data (CNIL number 1238249). The study was conducted in accordance with the Declaration of Helsinki.

### Xenogeneic Transplantation

NOD.Cg-Prkdc*^scid^*IL2RG*^tm1wjl^*/SzJ (005557) mice known as NOD scid gamma (NSG) mice (Jackson Laboratory, Bar Harbor, MI) were housed in the pathogen-free animal facility of Institut Universitaire d’Hématologie (Paris). Male or female NSG mice (n > 400) were xenografted at 2 months of age. Mice were irradiated with 2.25 Gy 24 h before injection of 0.3 × 10^7^ PBMCs in the caudal vein. Alternatively, CD4^+^CD8^−^ (10^6^ cell/mouse), CD4^+^CD8^+^ (0.3 × 10^5^ cell/mouse) or CD4^−^CD8^+^ TL (10^6^ cell/mouse) from healthy donor apheresis or the spleen xeno-GVHD mice were sorted with a BD FACS Aria III sorter (BD Biosciences; purity ≥ 95%) before injection to primary or secondary recipients. Mice were assessed for survival daily and weighed weekly. Development of aGVHD was monitored weekly using recently developed aGVHD scoring system based on binary evaluation (0 if absent; 1 if present) of weight loss (>10%), hunched posture, ruffled fur, skin lesions, diarrhea and reduced mobility (38). Diseased mice were sacrificed 4 to 6 weeks after transplantation when weight loss exceeded >15%. Mice blood was obtained by direct intra-cardiac puncture immediately before sacrifice. Femur, tibia, spleen, lungs, and liver were aseptically excised before being subjected to mechanical dissociation. Single-cell suspensions were filtered through a cell strainer (70 µm; BD Biosciences) and processed for flow cytometry analyses. The Ethical Committee at Paris Nord University approved all performed experiments.

### Flow Cytometry Analysis of Blood TL From Transplanted Patients

Flow cytometry analysis of TL subsets from 40 GVHD and non-GVHD transplanted patients, as well as of seven healthy blood donors were performed as described below. Sibling-identical donor transplantations were performed in 40 patients. Main stem cell source was GCSF-mobilized peripheral blood stem cells (PBSC) (63%), whereas BM was used in 19 patients (37%). GVHD prophylaxis based on cyclosporine at the dose of 3 mg/kg combined with either methotrexate (15 mg/m^2^ day 1, 10 mg/m^2^ days 3, 6, and 11) for patients receiving myeloablative conditioning, or mycophenolate mofetil (30 mg/kg/day) for patients receiving reduced-intensity conditioning. Main characteristics of allo-HSCT patients are summarized in **Table S2**. This study was performed in compliance with the Declaration of Helsinki after approval by the ethics committee of the Hospital Saint Louis (Paris, France). Written informed consent was obtained from all participants. Most patients were treated for hematological malignancies.

### Confocal Laser Imaging of Duodenal and Colic Biopsies From Transplanted Patients

Serial frozen sections (5-µm-thick) of rectal biopsies from 12 allo-HSCT patients with clinical evidence of aGVHD or control transplanted patients developing intestinal symptoms but without clinical GVHD were mounted on Superfrost Plus slides (Fisher Scientific; 160 serial sections/biopsy). Main characteristics of allo-HSCT patients are summarized in **Table S3**. After thawing at room temperature (45 min) sections were incubated for 10 min in PBS 1×, permeabilized in PBS 0.5% Triton (15 min) and incubated in blocking solution (PBS 3% BSA, 0.2% Tween-20; 30 min) before overnight incubation at +4°C with rat anti-CD8 (Abcam, ab60076), rabbit anti-CD4 (Abcam, ab133616) and mouse anti-CD3 (Agilent, M7254) (all 1/100 dilution) primary antibodies. After washing in PBS 0,1% Tween-20 (5 min) sections were incubated with donkey anti-rat Alexa488 conjugated (Invitrogen, A21208), donkey anti-Rabbit Alexa568 conjugated (Invitrogen, A10042) and donkey anti-mouse Cyanin5 conjugated (Jackson Immunoresearch, 715-175-151) (1/200 dilution). After washing sections were counterstained with DAPI (10 µg/ml; 1 h at room temperature), washed again in PBS 0.1% Tween-20 and mounted with Fluoromount-G medium (Thermo-Fischer, 00-4958-02). Negative controls were tissue sections incubated with concentration-matched secondary antibodies. Sections were scanned at high resolution before assembly of individual fields. Image montages were constructed using the ZEN software (Zeiss).

### Flow Cytometry and Cell Sorting

Single-cell suspensions were incubated with human Fc receptor-binding inhibitor (Fc Block, eBioscience) before staining with anti-human monoclonal antibodies (mAbs). Fluorescence minus one (FMO) controls with isotype controls were used to define positive signals for flow cytometry or cell sorting. Dead cells were excluded with the Zombie Violet Fixable Viability Kit (Biolegend). For labeling cells were resuspended in PBS, 2% FCS (1 to 5 × 10^7^ cells/500 μl) and incubated with the following mAbs: CD45 APC-Cy7/AF700/APC (Biolegend, clone HI30), CD19 APC/PE-DAZZLE/BV711 (Biolegend, clone HIB19), CD33 PE-CF594 (BD Biosciences, clone WM53), CD14 PC7/APC (Biolegend, clone M5E2), CD56 PC7 (Biolegend, clone MEM-188), CD57 BV605 (BD Biosciences, clone NK-1), CD94 APC/FITC (Biolegend, clone DX22), CD3 BUV395/BUV737/PE-CF594 (BD Biosciences, clone HUCHT1), CD4 APC-Cy7/BV395/PE (BD Biosciences, clone RPA-T4), CD8a AF700/BV421/PC7 (Biolegend, clone RPA-T8), CD8b APC (BD Biosciences clone 2ST8.5H7), CD95 BV605/PE (Biolegend, clone DX2), CD27 BV510 (Biolegend, clone M-T271), CD28 PE/PE-DAZZLE (Biolegend, clone CD28.2), CD45RA BV711/PE (Biolegend, clone HI100), CD45RO BV711 (Biolegend, clone UCHL1), CD127 PC5 (Biolegend, clone A019D5), CD194/CCR4 BV605 (Biolegend, clone L291H4), CD122 APC/PC7 (Biolegend, clone TU27), ITGB7 PC7 (eBioscience, clone FIB504), CD197/CCR7 PE/BV785 (Sony, Clone GOA43H7), CD199/CCR9 PE/PC7 (Biolegend, clone LO53E8), CD183/CXCR3 BV711 (Biolegend, clone G025H7), CD184/CXCR4 PC5 (Biolegend, clone 12G5), CD185/CXCR5 PE-DAZZLE (Biolegend, clone J252D4), CD186/CXCR6 APC (Biolegend, clone K041E5), CD103 PE/BV421 (Biolegend, clone Ber-ACT8), CD279/PD-1 PerCPeFluor 710 (eBiosciences, clone eBioJ105), CD62L APC/FITC (Biolegend, clone GREG-56), CD69 BV650 (Biolegend, clone FN50), KLRG1/CLEC15A BV605 (Biolegend, clone 2F1/KLRG1). Intracellular staining was performed using the Foxp3 staining buffer kit (eBioscience) with the following mAbs: Granzyme B FITC (Biolegend, clone GB11), Perforin APC (Biolegend, clone dG9), RORC APC (eBioscience, clone ASKJS-9), Eomes PE (eBioscience, clone WD1928), T-bet BV421 (Biolegend, clone 4B10), FOXP3 PE (Biolegend, clone 206D), GATA3 APC (Biolegend, clone 16E10A23), BLIMP-1 AF647 (BD Biosciences, clone Sanquin-Hobbit/1), Ki67 (Biolegend, clone Ki67).

Flow cytometry and cell sorting experiments were performed with a BD Fortessa Analyzer or a BD FACSAria III sorter (BD Biosciences; (purity ≥ 95%). Flow cytometry analyses were performed using the FlowJo software (Version 10.4) and the Spade algorithm (39).

### Fluidigm Analyses

Gene expression analyses were performed with the Fluidigm 96.96 Dynamic Array IFC and TaqMan Gene Expression Assays (Life Technologies). Fifty cells were sorted directly into 96-well PCR plate containing 2.5 μl TaqMan specific gene assay mix (Applied Biosystems), 5 μl of CellsDirect 2× Reaction mix, 0,2 μl SuperScript™ III RT/PlatinumR Taq Mix (Invitrogen, CellsDirect one-step qRT-PCR kit), 1.2 ml TE buffer, and 0.1 μl SUPERase-In RNase Inhibitor (Ambion). Reverse transcription was performed for 15 min at 50°C followed by 2 min at 95°C for RT inactivation. The corresponding cDNAs were then preamplified for 21 cycles at 95°C for 15 s and 60°C for 4 min. Preamplified products were diluted 1:5 in TE buffer and analyzed on a Biomark system (Fluidigm) with the following PCR cycling condition: 95°C for 10 min and 40 cycles at 95°C for 15 s and 60°C for 60 s. Data were analyzed using the Biomark qPCR analysis software (Fluidigm). For gene expression quantification, data were exported as an Excel file and analyzed by the ΔΔCt method. Results were normalized to HPRT or GAPDH and expressed as means of 3 to 12 biological replicates. Hierarchical clustering was performed on standardized means of gene expression levels with the Euclidean or the Manhattan distance of the “pheatmap” R package. References of the TaqMan primers used for the analysis are provided as **Table S1**.

### Multiplex Assay for Measurement of Cytokine Production

To assess cytokine production CD4^+^CD8^−^, CD4^+^CD8^+^ and CD4^−^CD8^+^ TL lymphocytes (5 * 10^3^ cells/well) sorted by FACS from the spleen of xenografted mice (4–6 weeks post-transplant) were stimulated for 5 h with phorbol 12-myristate 13-acetate (PMA; 20 ng/ml) and ionomycin (500 ng/ml) (both from Sigma-Aldrich). Cell-free supernatants were then collected and cytokine concentrations (IL-2, IL-4, IL-5, IL-6, IL-9, IL-10, IL-13, IL-17A, IL-17F, IL-22, IFN-γ, and TNF-α) were measured using the LEGENDplex™ human Th Panel (12-plex; BioLegend) system according to the manufacturer’s instructions.

### Assessment of Cytokine Expression by TL From Skin Infiltrates

Skin biopsies obtained with inform consent from healthy donors undergoing plastic surgery, from a patient with psoriasis or from 2 patients with cGVHD were performed under sterile conditions. Skin pieces were cultured in RPMI 1640 supplemented with fetal calf serum (10%), human AB serum (5%) and low doses IL-2 (60 Units/ml) with half medium change and IL-2 renewal every day. On culture-day 9, emigrant TL were harvested, washed and seeded for 5 h in 96-well plate with phorbol-myristate-acetate (10 ng/ml; Sigma), ionomycin (1 μg/ml; Life Technologies), brefeldin A (10 μg/ml; Sigma), and Monensin (BD GolgiStop™; 1/1000, BD Biosciences). Cell were then processed for permeabilization (Foxp3 staining buffer kit, eBioscience) and labeled with the following antibodies: CD3 V500 (clone UCHT1, Beckman Coulter), CD4 PerCP-Cy5 (clone RM4-5, eBioscience), CD8 FITC (clone 53–6.7, eBioscience), IL-17 APC (clone eBiol 7B7, eBioscience), IL-10 AF405 (clone JES-19F1, Biolegend), γ-IFN PE (clone B27 RUO, BD Biosciences). Flow cytometry analyses were performed as above with a BD Fortessa Analyzer and the FlowJo software (Version 10.4).

### Statistics

Statistical analysis was performed using GraphPad Prism version 7. Significance was assessed using a Mann–Whitney U test for paired comparisons or a Kruskal-Wallis test for multiple comparisons. A P value ≤ 0.05 was taken to indicate a significant difference between groups. Asterisks indicate statistical significance (p < 0.0001****; p < 0.001***; p < 0.01**); when p is comprised between 0.05 and 0.01 the exact value in indicated in the figures.

## Results

A major limitation of clinical studies based on analysis of circulating TL in human HSCT recipients developing aGVHD is to what extent they reflect the phenotypic and functional diversity of alloreactive T cells within aGVHD target organs. As a goal to bypass this constraint and to further characterize tissue-infiltrating TL we developed a model of xenogeneic GVHD based on intravenous injection of hu-PBMCs from healthy donors into NSG mice. Consistent with earlier reports (40–43) preliminary experiments found that irrespective of the cell dose (0.3–1 × 10^7^ cells/mouse) ≥80% engrafted mice develop symptoms of aGVHD within 6 weeks after transplant. Since the number of injected cells affected neither the delay of aGVHD onset nor disease severity, subsequent experiments were performed with the lowest dose, i.e. with 0.3 × 10^7^ PBMC/mouse. Comparison between irradiated (n = 24) and non-irradiated (n = 11) mice infused with hu-PBMC subsequently showed that, despite overall similar TL engraftment and expansion, the latters developed a milder form of the disease characterized by decreased weight loss (median weight: 29 *vs.* 23 g) and slightly delayed aGVHD onset (median: 1.2 week). This confirms that the inflammatory context caused by radiation-induced tissue damages affects xenoreactive hu-TL activation.

**Figure 1:**
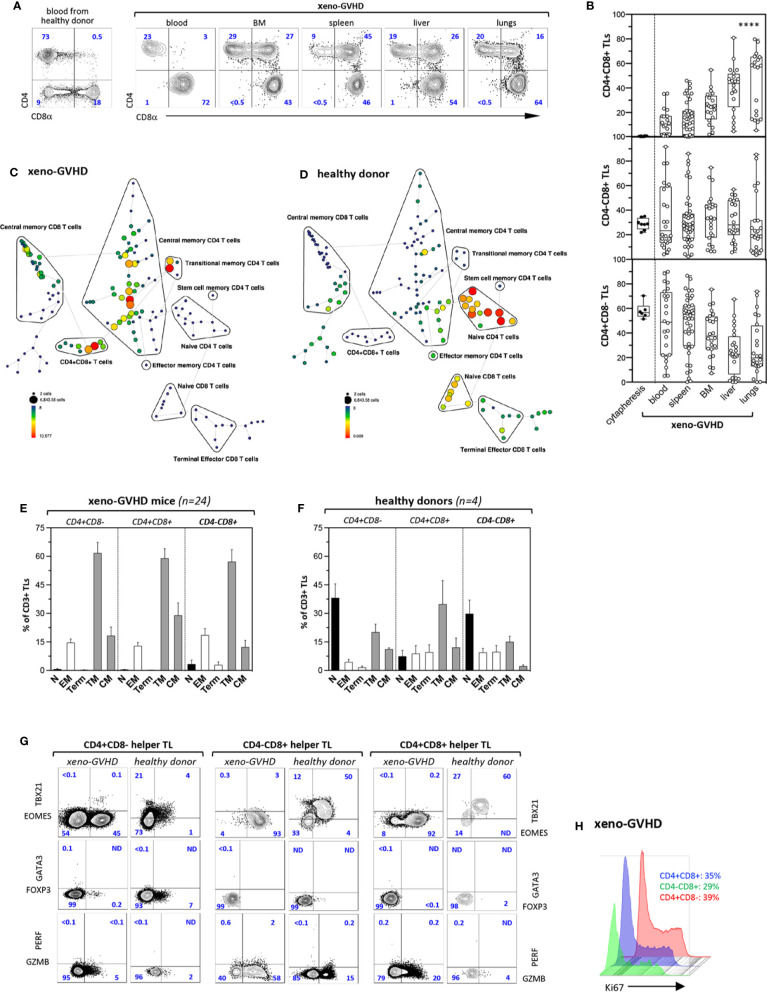
Xenogeneic GVHD induce tissue accumulation of CD4^+^CD8^+^ memory TL. **(A)** Phenotype of CD3^+^ TL isolated from the blood of a healthy donor (left panel) or from the blood and aGVHD target organs of a diseased mouse (right panels). Sub lethally irradiated mice injected with 3 × 10^6^ PBMCs were sacrificed 4 to 6 weeks later (weight loss was >15%). Gates are set on human (hu) CD45^+^CD3^+^ TL; contour plots show percentages of the CD4^+^CD8^−^, CD4^−^CD8^+^, and CD4^+^CD8^+^ subsets. **(B)** Distribution of CD4^+^CD8^−^, CD4^−^CD8^+^, and CD4^+^CD8^+^ hu-TL in blood (n = 23), spleen (n = 36), BM (n = 25), liver (n = 27), and lungs (n = 23) of diseased mice. Diseased mice were sacrificed 4 to 6 weeks after transplantation. After staining for surface antigens cells were analyzed by flow cytometry. Box and whisker plots display the median, 25th, and 75th percentile of the distribution, and whiskers extend to the most extreme data points. Black and empty circles correspond respectively to individual donor apheresis or diseased mice. Data are compiled from four independent experiments. Data are compiled from 4 independent experiments. Asterisks indicate statistical significance based on the Kruskall-Wallis test (p < 0.0001****). **(C, D)** Tree plot representation showing immunophenotypic stratification of TL subsets isolated from the **(C)** the spleen of a representative aGVHD mouse injected with PBMC from **(D)** the blood of a healthy donor. Down sampling and clustering analysis based on expression of 16 cell-surface markers (CD4, -8a, -45RA, -25, -27, -28, -56, -62L, -69, -95, -103, -122, -127, -197, -199, -279) was performed using the Spanning-tree Progression Analysis of Density-normalized Events (SPADE) algorithm. The size and color of each circle indicate the frequency of cells. Populations of interest were annotated manually and represented as black lines encircling the corresponding set of nodes. **(E, F)** Immunophenotypic stratification of hu-TL subpopulations circulating in the blood of **(E)** aGVHD mice or **(F)** from the corresponding healthy donor. Gates were set as shown in panels A and C. Bars represent mean percentages ± standard deviation of the indicated populations. Results are from 4 healthy donor apheresis and 24 xeno-GVHD mice. **(G)** Expression of transcription factors EOMES, TBX21/TBET, GATA3 and FOXP3, as well as of cytolytic markers PERF and GZB by CD4^+^CD8^−^, CD4^−^CD8^+^, and CD4^+^CD8^+^ TL subsets from the spleen of a diseased mouse (*left columns*) transplanted with PBMC from the same healthy donor (*right columns*). Cells were fixed, permeabilized and labeled with an eight-color antibody panel. Bi-dimensional contour plots show expression of the indicated markers. Percentages of the corresponding populations are indicated. **(H)** Histograms show expression of proliferation marker Ki-67 by hu-TL from the spleen of a diseased mouse. Cells were processed as indicated above. Percentages of Ki-67–positive cells are indicated.

Flow cytometry analyses of human leukocytes isolated from the blood or tissues of diseased mice found that CD3^+^ TL accounted for ≥98% of huCD45^+^ cells while detecting only rare CD33^+^CD14^−/+^ monocyte/granulocytic cells (data not shown). Further characterization of hu-TL circulating in the blood of diseased mice disclosed selective expansion of a minor CD4^+^CD8^+^ (DP) TL subset whose percentages increased about 10-fold relative to those observed in donor blood (median: 11% [n = 22] *vs.* 0.7% [n = 6]) (**Figures 1A, B**). Consistent with these results, DP TL were also detected at a higher frequency in aGVHD target organs accounting for up to 45% of hu-T cells in the spleen (median: 15%, n = 34) or BM (median: 23%, n = 24) and reaching 80% in the liver (median: 37%, n = 25) or lungs (median: 30%, n = 23). It should also be noted that percentages DP TL in blood or in GVHD target organs were not affected by prior irradiation. Similarly, no changes in DP TL percentages were observed according to T cell expansion as assessed by human-mouse chimerism, delay to disease onset or aGVHD clinical score (data not shown).

Immunophenotypic stratification using the SPADE algorithm analysis followed by tree plot representation of clustered expression of 16 surface markers (39) found that, irrespective of their CD4^+^CD8^−^, CD4^−^CD8^+^, or CD4^+^CD8^+^ DP phenotype, hu-TL undergo a major phenotypic transition toward a transitional/central memory phenotype (CD27^+^CD28^+^CD45RA^−^CCR7^−/+^) resulting in almost complete disappearance of the naive compartment (**Figures 1C, D**). Manually curated stratification of blood CD4^+^CD8^−^, CD4^−^CD8^+^, or DP TL from 24 diseased mice and 4 healthy donors into naïve (N: CD27^+^CD28^+^CD45RA^+^CCR7^+^), transitional (TM: CD27^+^CD28^+^CD45RA^−^CCR7^−^), effector (EM CD4^+^/CD4^+^CD8^+^: CD27^−^CD28^+^; EM CD8^+^: CD27^+^CD28^lo^) or central memory (CM: CD27^+^CD28^+^CD45RA^−^CCR7^+^) and terminally differentiated (Term: CD27^−^CD28^−^) subsets (44) largely confirmed these results (**Figures 1E, F**). Analysis of DP TL circulating in the blood of healthy donors found in addition that they comprise a majority (>80%) of antigen-experienced CD45RA^−^ TL which is consistent with a previous report (45).

Further phenotypic characterization of blood and tissue-infiltrating hu-TL from diseased mice based on expression of a series activation/maturation markers (CD69, KLRG1, CD95, PD-1/CD279), adhesion molecules (CD56, CD62L, CD103), CXC (CXCR3/CD183, CXCR4/CD184, CXCR5/CD185, CXCR6/CD186) or CC (CCR4/CD194, CCR5/CD195, CCR6/CD196, CCR7/CD197, CCR9/CD199) chemokines receptors, as well as of diverse cytokine receptors (CD25, CD122, CD127) found that irrespective of their origin, lineage affiliation or differentiation status they expressed low CD183/CXCR3 levels known to be associated with a T_H_1 polarization (46) but remained negative for expression of chemokine receptors known to be expressed by the T_H_2 (CD194/CCR4) or TFH (CD185/CXCR5) subsets. Consistent with their effector/memory phenotype and, here again, irrespective of lineage affiliation, xenoreactive hu-TL expressed high CD95/Fas levels. Whereas only a min fraction (<2%) of tissue-infiltrating hu-TL stained positive for CD103/ITGAE a marker of tissue resident memory CD8^+^ TL (47), > 80% of them expressed CD279/PD-1 from which about 30% were CD62L^+^CD69^+^. Notably, a minor CD25^+^ subset (1–2%) likely corresponding to regulatory-T cells was also detected among CD4^+^ helper TL (data not shown).

Hu-CD45^+^ TL from the spleen of diseased were then tested for expression of a series of nuclear (Eomes, TBX21, GATA3, FOXP3, Ki67) or cytoplasmic (Granzyme B/GZMB, Perforin/PERF) markers, and compared to their blood counterparts from the same donor. This led to finding that persistent activation increases by 10-fold percentages of Eomes^+^ cells within CD4^+^CD8^−^ helper TL from which a min fraction, likely corresponding to T_H_1 cells, retained high TBX21/Tbet expression levels (**Figure 1G**, left panel). Helper T cells also included rare GATA3^+^ T_H_2 or FOXP3^+^ regulatory-T cells and 5% to 10% of them acquired cytoplasmic GZMB (48) indicating that they retain a significant degree of functional diversity. On the other hand, persistent activation of CD4^−^CD8^+^ CTL led to homogeneously high Eomes expression, almost complete loss of TBX21/Tbet, as well as to a three-fold increase in percentages of GMZB^+^ cells (**Figure 1G**, medium panel). Analysis of CD4^+^CD8^+^ DP TL found that their phenotypic profile largely overlapped that of CD4^−^CD8^+^ CTL which was suggestive of close developmental relationship between the two (**Figure 1G**, right panel). CD4^+^CD8^+^ DP TL lacked FOXP3 which rules out that they might correspond to peripherally-derived regulatory TLs. Finally, that about one-third of chronically activated CD4^+^CD8^−^, CD4^−^CD8^+^ or DP spleen T cells expressed Ki67 further indicates that xenoreactive TL undergo massive *in situ* polyclonal amplification in aGVHD organs (**Figure 1H**).

Collectively, these results show that the development of aGVHD in xenografted mice is associated with a massive switch of hu-TL toward an activated transitional memory profile, as well as with expansion of an original CD4^+^CD8^+^ DP TL subset. That irrespective of their origin and lineage affiliation xenoreactive hu-TL display highly monotonous immunophenotypic landscapes indicates in addition that tissue-specific environment does not exert a major influence on TL activation and/or maturation in this context.

**Figure 2:**
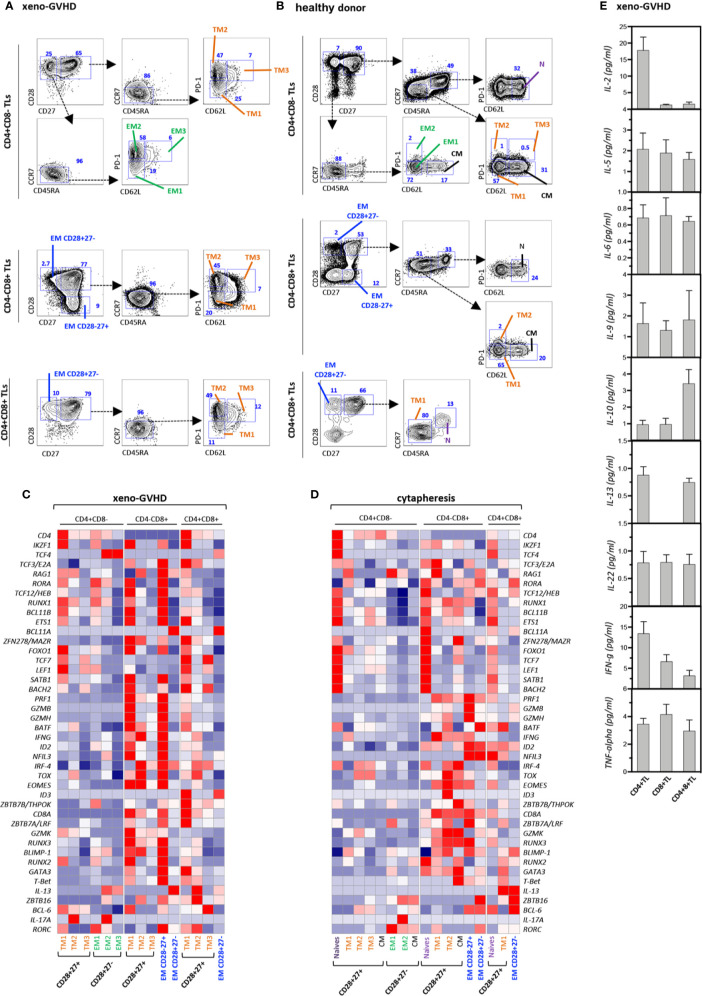
Gene expression profiling and cytokine production of resting and xenoreactive TL. **(A, B)** Gating strategy to subdivide **(A)** xenoreactive and **(B)** resting CD4^+^CD8^−^, CD4^−^CD8^+^ or DP TL from the same donor. Mouse splenocyte or hu-PBMC were labeled with a 10-color antibody panel before CD4^+^CD8^−^, CD4^−^CD8^+^, or DP hu-TL were partitioned into 15 populations or 17 cellular fractions based on differential CD27, CD28, CD45RA, and CCR7 expression. The gating procedures defining the naïve (N), central memory (CM), transitional memory (TM1–3), effector memory (EM) and terminally differentiated (Term) subsets are indicated. Bi-dimensional contour plots show expression of the indicated markers. Arrows indicate the gating hierarchy. Percentages of the corresponding populations are indicated. **(C, D)** Heatmaps show expression of a set of 42 genes by 15 populations of **(C)** xenoreactive or **(D)** resting. Pools of 50 cells were sorted and analyzed by multiplex RT-PCR. Gene expression levels normalized relative to HPRT correspond to mean of 3 to 12 replicates. **(E)** Assessment of cytokine production in culture supernatants. CD4^+^CD8^−^, CD4^+^CD8^+^, or CD4^−^CD8^+^ TL sorted by FACS from the spleen of diseased mice at week 4 after grafting were stimulated for 5 h with PMA and ionomycin. Results are expressed in pg/ml. Data are means and SD of 5 replicates from one representative experiment.

To get further insight in their origin and function, CD4^+^CD8^+^ TL as well as conventional helper CD4^+^CD8^−^ and cytotoxic CD4^−^CD8^+^ TL from the spleen of diseased mice or the blood of a healthy donor were fractionated based on differential CD27, CD28, CD45RA, CCR7, PD-1, and CD62L expression into N, TM1–3, EM and CM subsets (**Figures 2A, B**), and analyzed by multiplex PCR for expression of 46 regulators or classifiers of T cell differentiation and function (**Table S1** and **Figures 2C, D**). Gene expression analyses confirmed co-expression of *CD4* and *CD8a* transcripts by the DP subset indicating that their combined detection by FACS does not result from non-specific labeling or membrane shedding. Side by side comparison showed that, although partially overlapping, transcriptional signatures of resting and chronically activated TM1 CD4^+^CD8^+^ TL differed from those of their CD4^−^CD8^+^ CTL counterparts by decreased levels of cytotoxicity-related *PRF1*, *GZMB*, *GZMH*, and *GZMK* transcripts. Relative to CD4^−^CD8^+^ CTL, CD4^+^CD8^+^ TM1 also down modulated transcripts coding *RUNX3*, *Eomes*, and *T-bet* transcription factors involved in CD4 silencing and reciprocal activation of a cytotoxic program, while they expressed at higher levels CD4 transcriptional activators, *ZBTB7B/THPOK* and *ZBTB7A/LRF* (49). Interestingly, TM1 DP TL also expressed decreased IFNγ transcript levels. Upregulation of PD-1 by DP TM2/3 TL coincided with transcriptional remodeling characterized by shutdown of the cytotoxic transcriptional program and corresponding upregulation of immunoregulatory IL-13. Finally, regardless of their CD4^+^CD8^−^, CD4^−^CD8^+^, or CD4^+^CD8^+^ affiliation, expression of CD62L expression by PD1^+^ TM3 T cells was accompanied by further down-modulation of most transcripts suggestive of functional exhaustion.

CD4^+^CD8^+^ DP as well as single positive CD4^+^ helper and CD8^+^ cytotoxic TL sorted from the spleen of diseased mice were then activated for 5 h with PMA and ionomycin before quantification of cytokine production in culture supernatants. This led to finding that, consistent with their transcriptional signature, DP TL produce IL-10 and IL-13 at the highest levels, while they retain only marginal capacity to secrete IL-2 and IFN-γ (**Figure 2E**).

**Figure 3:**
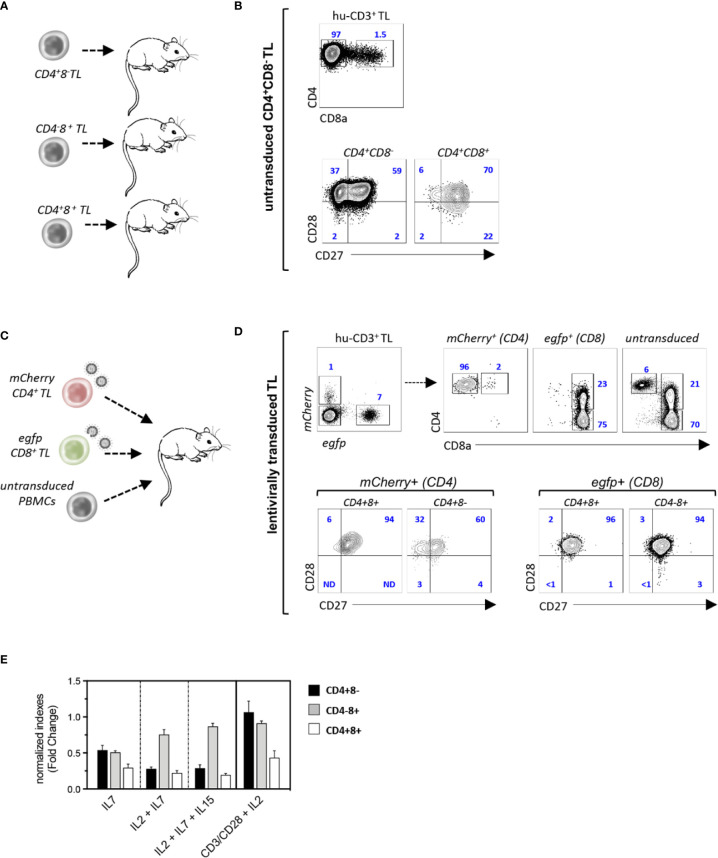
CD4^+^CD8^+^ TL differentiate from chronically activated CTL. **(A, B)** Experimental design: **(A)** CD4^+^CD8^−^, CD4^+^CD8^+^, or CD4^−^CD8^+^ TL (purity: 98%) from a healthy donor were injected to irradiated NSG mice (CD4^+^CD8^−^/CD4^−^CD8^+^: 10^6^ cells/mouse; CD4^+^CD8^+^: 10^5^ cells/mouse; n = 3); recipient mice were sacrificed 4 weeks after grafting and spleen hu-TL were subjected to FACS analysis. **(B, C)** Contour plots show expression of CD8α (eft panel) and CD27/CD8 (right panels) by xenoreactive T cells from one representative mouse injected with CD4^+^CD8^−^TL out of 5. **(C, D)** Experimental design: **(C)** CD4^+^CD8^−^ or CD4^−^CD8^+^ TL (10^6^ cells each/mouse) activated overnight with CD3/CD28-coated nanobeads in the presence of IL-2 before exposure to mCherry (CD4^+^) or egfp (CD8^+^) reporter viruses were mixed with untransduced PBMC from the same donor (10^6^ cells/mouse) before injection to NSG mice (n = 3). Spleen hu-CD3^+^ TL from mice at week 4 after grafting were analyzed as above. **(D)** Upper contour plots: engraftment efficiencies and CD4 or CD8 co-receptors expression by helper (*mCherry*^+^), cytotoxic (*egfp*^+^) and control untransduced TL; lower contour plots: expression of CD27/CD28 by *mCherry*^+^ or egfp^+^ CD4^-/+^CD8^-/+^ T cells. **(E)** CD4^+^CD8^-^, CD4^+^CD8^+^ or CD4^-^CD8^+^ TL (104 cells/well) isolated from the spleen of diseased mice were cultured for 96 hours with IL-2, IL-7 or IL-15 (10 ng/mL each) and/or CD3/CD28-coated nanobeads. Results are expressed as normalized growth indexes. Data are means and SD of 3 replicates from one representative experiment.

Since these results suggested that CD4^+^CD8^+^ TL differentiate from chronically activated CTL, NSG mice were then transplanted with purified CD4^+^CD8^−^, CD4^−^CD8^+^ or CD4^+^CD8^+^ TL sorted from a healthy donor apheresis (**Figure 3A**). Phenotypic characterization of spleen hu-CD45^+^ TL 4 weeks later found that only CD4^+^CD8^−^ helper TL efficiently engrafted recipient mice in which they could then represent up to 40% of splenocytes. However, despite engraftment, only a minority of them (< 2%) corresponding to CD27^+^CD28^+^ TM cells could acquire surface CD8α (**Figure 3B**). To substantiate these findings secondary transfers of CD4^+^CD8^−^, CD4^−^CD8^+^, or CD4^+^CD8^+^ hu-TL sorted from the spleen of diseased mice were then performed which, here again, led to finding that only CD4^+^CD8^−^ TL engraft secondary recipients albeit with limited efficiency (2–3%), from which only a minority (0.5–1%) acquired surface CD8α (data not shown).

Since the lack of engraftment of CD4^−^CD8^+^ CTL could relate to low IL-2 production levels, an *in vivo* tracking strategy based on lentiviral-mediated genetic labeling was then devised to evaluate their capacity to differentiate into CD4^+^CD8^+^ TL. Single positive CD4^+^CD8^−^ helper and CD4^−^CD8^+^ CTL from a healthy donor were therefore transduced overnight with *mCherry* (CD4^+^) or *egfp* (CD8^+^) reporter viruses and mixed with unfractionated autologous PBMC before infusion to irradiated NSG mice (**Figure 3C**). Characterization 4 weeks later of spleen hu-TL showed efficient engraftment of both TL subsets under these conditions and found, as expected, a higher expansion of the CD8^+^ CTL (7- to 10-fold). Phenotypic characterization of *mCherry*^+^-CD4^+^ TL found, here again, that only a few of them (1–2%) had acquired surface CD8α. In contrast, 20% to 30% of their *efgp*^+^-CD8^+^ counterparts expressed high membrane CD4 levels, from which a majority corresponded to PD1^+^ TM cells (**Figure 3D** and data not shown). To search for subset-specific growth/survival requirements, xenoreactive CD4^+^CD8^−^, CD4^−^CD8^+^, and CD4^+^CD8^+^ hu-TL sorted from the spleen of diseased mice were then seeded for 96-h with various combinations of γc cytokines (IL-2, IL-7, IL-15) or CD2/CD28 coated nanobeads (**Figure 3E**). Although TCR or γc signaling improved the yield of CD4^+^CD8^−^ or CD4^−^CD8^+^ TL, they had only limited effect on CD4^+^CD8^+^ DP TL. Taken as a whole, these data show that a vast majority of CD4^+^CD8^+^ TL differentiate from chronically activated CD8^+^ CTL undergoing functional diversion to a terminally differentiated and possibly exhausted immunoregulatory phenotype.

**Figure 4:**
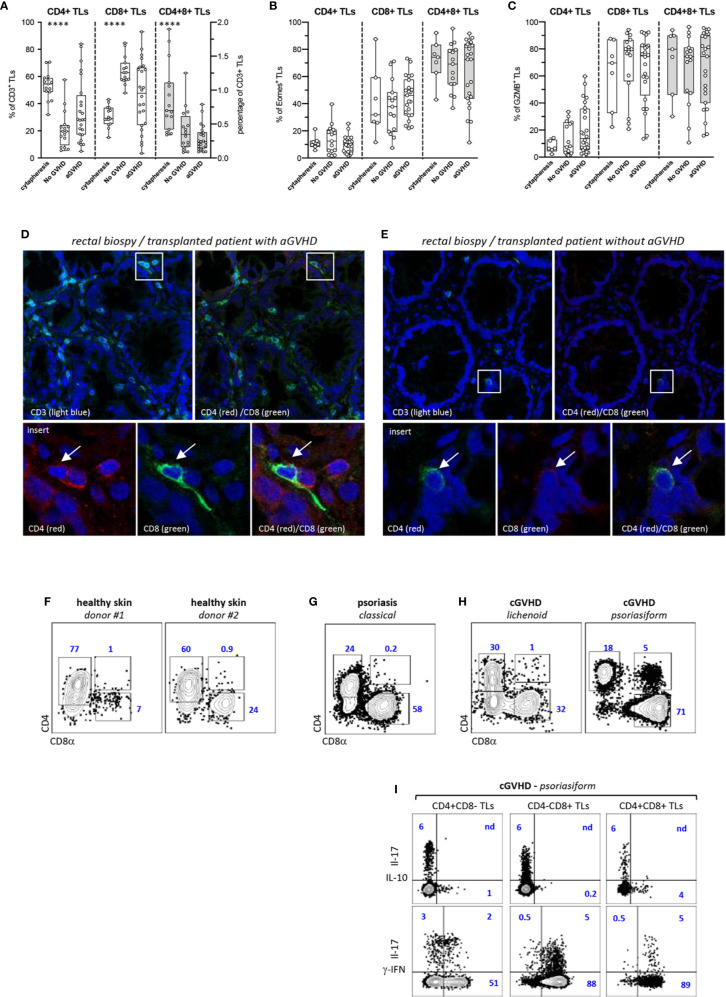
Decreased proportions of CD4^+^CD8^+^ TL in the blood allo-HSCT patients. **(A–C)** Phenotypic characterization of TL circulating the blood of allo-HSCT patients. **(A)** Proportion of CD4^+^CD8^−^ (CD4^+^), CD4^−^CD8^+^ (CD8^+^), and CD4^+^CD8^+^ T-cell subpopulations within CD3^+^ TL in 15 healthy donors and a series allo-HSCT patients with (n = 24) or without (n = 16) aGVHD. Box and whisker plots display the median, 25th, and 75th percentile of the distribution (box), and whiskers extend to the most extreme data points. Gray boxes show distribution of the CD4^+^CD8^+^ T-cells. Circles correspond to individual donors or allo-HSCT patients. **(B, C)** Expression of **(B)** eomes or **(C)** granzyme B (GZMB) by corresponding T-cell subpopulations. Asterisks indicate statistical significance based on the Kruskall-Wallis test (p ≤ 0.0001****). **(D, E)** Analysis by high-resolution fluorescence of rectal biopsies from allo-HSCT patients **(D)** with or **(E)** without aGVHD. Tissue sections were scanned a high resolution before assembly of individual fields. Lower panels show higher magnification of corresponding insets. Arrows indicate tissue infiltrating double positive (CD3^+^) CD4^+^CD8^+^ TL. **(F–I)** Phenotypic characterization of skin TL from **(F)** 2 healthy donors, **(G)** a psoriasic patient and **(H)** two transplanted patients with lichenoid or psoriasiform cGVHD lesions. Gates are set on CD3^+^ TL; contours plots show relative percentages of CD4^+^CD8^−^, CD4^−^CD8^+^ and CD4^+^CD8^+^ TL. **(I)** IL-17, IL-10, and γ-IFN expression by TL subsets isolated from psoriasiform cGVHD skin lesions; contour plots show percentages of positive cells.

We then investigated whether CD4^+^CD8^+^ DP TL could also participate in the control of aGVHD in human HSCT recipients. Phenotypic characterization of blood CD3^+^ TL in a cohort of 40 transplanted patients matching for age, sex, disease, conditioning regimen and GVHD prophylaxis showed, here again, preferential expansion of donor-derived CD4^−^CD8^+^ CTL (**Table S2**) (**Figure 4A**). Side-by-side comparison between patients with (n = 24) or without aGVHD (n = 16) found higher percentages of CD4^+^CD8^−^ helper TL (median: 28% vs. 19%) as well as corresponding decrease of CD4^−^CD8^+^ CTL (median: 45% vs. 63%) in those developing aGVHD (p<0.04 by the Mann-Whitney test). Percentages of DP T cells followed a similar trend since they dropped from a median of 0.7% in healthy donor apheresis (n = 15) to 0.35% and 0.23% in allo-HSCT recipients with or without aGVHD. Further immunophenotypic stratification confirmed that irrespective of aGVHD development and TL lineage affiliation homeostatic proliferation induces a massive switch of CD3^+^ TL toward a memory profile (data not shown). Subsequent analyses showed, as expected, that Eomes and GZMB expression remained largely confined to CTL and DP TL but, here again, no difference was observed according to aGVHD development (**Figures 4B, C**).

Inasmuch as these results suggested either that DP T cells undergo limited homeostatic proliferation or that they undergo rapid blood extravasation in allo-HSCT patients, a series of duodenal or rectal biopsies from 12 transplanted patients with (n = 6) or without (n = 6) aGVHD (**Table S3**) were then comparatively analyzed for the presence of DP TL. Confocal microscopy analysis disclosed a diffuse mucosal infiltration by alloreactive CD8^+^ TL in those patients developing aGVHD but found that only a min fraction (<1/500) of them co-expressed CD4 (**Figure 4D**). In contrast, only scarce CD3^+^ TL were detected in transplanted patients without aGVHD, from which those from the CD8^+^ subset could also express CD4, suggestive of possible involvement in maintenance of intestinal homeostasis (**Figure 4E**).

Since these results ruled out possible involvement of DP TL in the pathogenesis of human aGVHD, and inasmuch as it has been reported that this population is detected in the blood and tissues of patients with chronic inflammatory diseases, an exploratory study was carried out to determine whether tissue DP TL could be amplified in the cGVHD, and if so whether their cytokine production profile was also skewed toward IL-10. Flow cytometry analysis of TLs isolated from the skin of 2 healthy donors, from a patient with psoriasis, and from 2 transplanted patients with cGVHD disclosed abnormal expansion of DP TL only in the 47-year-old patient who developed a rare psoriasiform cGVHD with highly inflammatory skin lesions massively infiltrated by CD4^−^CD8^+^ CTL (**Figures 4F–H**). However, assessment of cytokine expression showed that most of DP TL (≥90%) express IFN-γ which rules out possible regulatory diversion (**Figure 4I**).

## Discussion

In this report, we developed a xenogeneic model of aGVHD in immunodeficient NSG mice with the aim to characterize tissue-infiltrating pathogenic TL and to search for eventual organ-specific subsets. Consistent with earlier reports (40, 42, 43, 50), human TL efficiently engrafted recipient mice in which they underwent massive polyclonal expansion and complete naïve to memory phenotypic switch. However, compared with human GVHD in which a preferential homeostatic amplification of the CD8^+^ TL compartment is routinely observed, in xenografted mice ratios between CD4^+^ helper and CD8^+^ CTL remained unchanged. Instead, we found that development of aGVHD in xenografted mice is associated with the emergence of an original subset of DP TL whose percentages reach maximum levels in the liver and lungs suggesting that repeated contacts with foreign molecules from the outside world and/or site-specific inflammatory contexts could promote their selective recruitment, differentiation and/or amplification. Abnormal expansion of phenotypically similar DP TL subsets has already been reported in a variety of pathological situations accompanied by chronic inflammation and persistent TL activation. However, to date their helper or cytotoxic origin and functional characteristics, i.e. whether they display cytotoxic or immunoregulatory properties, remain a matter of debates.

Functional characterization of DP TL isolated from diseased mice disclosed a Tr1 regulatory profile with secretion of high anti-inflammatory and profibrotic cytokines including IL-10 and IL-13, and low IL-2 and IFN-γ. These results are consistent with earlier reports that CD4^+^CD8αβ^+^ TL infiltrating urological (51) or colorectal tumors (34), or the skin of some aGVHD patients (35) display overall similar Tr-1 cytokine production profile, and they are reminiscent of another report showing that helper TL-derived CD4^+^CD8α^+^ T cells from the colonic lamina propria of patients with inflammatory bowel disease secrete IL-10 (52). They also suggest that through limitation of the tissues damages caused by other xenoreactive TL subsets, DP TL should contribute to mitigate aGVHD severity in xenografted mice.

As to their helper or cytotoxic origin, adoptive transfer to immunodeficient mice of genetically labeled TL found that DP TL mainly differentiate from chronically activated CD8^+^ CTL. In contrast, only a marginal fraction (< 2%) of genetically labeled CD4^+^ helper TL upregulated CD8α. This fits with our flow cytometry data showing that DP TL homogeneously express the CTL-specific transcription factor EOMES, as well as with molecular analyses showing overexpression of cytotoxicity-related transcripts. Also consistent with a preferential CD8^+^ CTL origin, earlier reports have shown that about 10 to 25% of human CD8^+^ CTL upregulate surface CD4 after 24–72 h activation by anti-CD3/CD28 antibodies (37, 53, 54) or co-culture with allogeneic dendritic cells (55). That CD8^+^ CTL isolated from the umbilical cord blood are prone to upregulate CD4 (55) suggests in addition that such heterogeneity in CD8^+^ CTL response could relate to age-dependent differences in TCR activation thresholds and/or ontogeny-related changes in lineage plasticity (56).

Our observation that, conversely to their counterparts from diseased mice, DP TL isolated from the skin of a patient developing a rare aggressive psoriasiform cGVHD display a prototypic IFN-γ^+^ Tc1 profile, adds further complexity to this picture. These data indicate that upregulation of surface CD4 by CD8^+^ CTL does not necessarily coincide with functional diversion toward a regulatory phenotype. Also consistent with the view that CD4 behaves as an activation marker of CD8^+^ CTL, cross-linking CD4 on TCR-activated DP TL further increases IFN-γ production and promotes acquisition of surface FasL which is suggestive of co-stimulatory activity (53). Similar disconnection between heterologous co-receptor re-expression and helper *versus* cytotoxic functional profile is also observed within CD4^+^ helper TL. In this regard, our finding that about 40% to 50% of xenoreactive CD4^+^ (CD8^−^) helper TL express EOMES is suggestive of partial reprogramming toward the cytotoxic lineage. Similarly, a recent report demonstrates that, in human, acquisition of cytotoxic functions by CD4^+^ helper T cells occurs independently of CD8α upregulation (48). This contrasts with mouse data showing that upregulation of CD8α by CD4^+^ helper TL marks the acquisition of cytotoxic functions (36, 57) and points to major differences in lineage plasticity and phenotype/function relationships between human and mouse (49). Finally, it should also be noted that no abnormal expansion of DP TL have been reported so far in mouse models of aGVHD (7).

Collectively, our results demonstrate that in a xenogeneic setting upregulation of surface CD4 by chronically activated human CD8^+^ CTL is associated with a process of functional diversion toward an immunoregulatory profile that might ultimately lead to functional exhaustion. This idea is further supported by our finding that whereas acquisition of PD-1^+^ by TM2 DP TL is associated with upregulation of *TOX*, a key driver of CD8^+^ CTL exhaustion (58), subsequent upregulation of surface CD62L by the TM3 DP TL coincides with a global transcriptional shutdown. Also consistent with this view, DP TL sorted from the spleen of diseased mice fail to expand in response to γc cytokines and/or CD3/CD28 antibodies.

Analysis of a cohort of transplanted patients failed to disclose abnormal expansion of blood DP TL compartment, thus casting shadow on the pathophysiological relevance of that xenogeneic approach. We found instead that, irrespective of aGVHD development, homeostatic proliferation of donor T cells results in a major contraction of the DP TL compartment. Furthermore, that only rare DP TL were detected among the CD8^+^ TL infiltrating the intestinal mucosa of patients developing aGVHD rules out possible compartmentalization. These discrepant findings indicate that despite massive polyclonal amplification the mouse environment does not allow for optimal activation/maturation of xenoreactive TL. This assumption is reinforced by our observation that, contrary to their counterparts from blood of healthy donors, xenoreactive TL essentially lack expression of TBX1, a transcription factor known to play a central role in Th1-type helper T cell polarization, as well as in maturation and maintenance of CD8^+^ CTL effectors (59). Although the underlying mechanisms remain elusive, we speculate that these abnormalities could relate to defective T-cell priming by mouse APC, decreased affinity of human TCR for mouse MHC molecules and/or limited helper activity. In the same manner, one cannot exclude that the lymphoid tissues or deep organs of NSG mice may also interfere with activation and maturation of xenoreactive human TL.

Collectively, our data show that the xenogeneic setting used in this study does not recapitulate several key features of the human disease which represents an important limitation for preclinical applications. To get closer to the human disease the xeno-GVHD model needs therefore to be implemented. A first step toward this aim would be the development of HLA-transgenic immune deficient mice humanized for M-CSF, IL-3, GM-CSF, and TPO cytokines which would allow for better preservation of the monocyte/macrophage and dendritic cell compartment and more efficient T-cell activation/maturation (60). Further, by improving myeloid reconstitution, co-injection of syngeneic hematopoietic stem cells in these mice could also ameliorate presentation of mouse antigens and thus result in enhanced T-cell reactivity.

## Data Availability Statement

The raw data supporting the conclusions of this article will be made available by the authors, without undue reservation.

## Ethics Statement

The studies involving human participants were reviewed and approved by the Ethical Committee at Paris Nord University. The patients/participants provided their written informed consent to participate in this study. The animal study was reviewed and approved by Ethical Committee at Paris Nord University. No potentially identifiable human images or data is presented in this study.

## Authors Contributions

KAH designed and performed most experiments and wrote the paper; DM and LD performed the SPADE analyses and conducted the clinical studies; VB, SK, and EN conducted mouse studies; JDB and HB performed the skin biopsies analyses; BC and GS ensured the scientific supervision of the project and wrote the paper. All authors contributed to the article and approved the submitted version.

## Funding

This work was supported by the Institut National du Cancer.

## Conflict of Interest

The authors declare that the research was conducted in the absence of any commercial or financial relationships that could be construed as a potential conflict of interest.
