# Supplementary material for: CD4+CD8+ T-Lymphocytes in Xenogeneic and Human Graft-versus-Host Disease
Source: Front Immunol. 2020 Nov 24;11:579776. doi: 10.3389/fimmu.2020.579776 (PMC7732609; doi:10.3389/fimmu.2020.579776)
Supplement: Supplementary file 1 [file Table_1.docx]

**Table S1**. List of taqman probes (by alphabetical order).

| **Name** | **Description** | **Reference** |
| --- | --- | --- |
| BACH2 | Transcription regulator protein BACH2 | Hs00222364_m1 |
| BATF | basic leucine zipper transcription factor, ATF-like | Hs00232390_m1 |
| BCL11A | B-cell lymphoma/leukemia 11A | Hs01093197_m1 |
| BCL11B | B-cell lymphoma/leukemia 11B | Hs01102259_m1 |
| BCL6 | B-cell lymphoma 6 protein | Hs00153368_m1 |
| CD4 | CD4 antigen | Hs01058407_m1 |
| CD8A | CD8 alpha antigen | Hs00233520_m1 |
| CD8B | CD8 beta antigen | Hs00174762_m1 |
| EOMES | Eomesodermin also known as T-box brain protein 2 | Hs00172872_m1 |
| ETS1 | Protein C-ets-1 | Hs00428293_m1 |
| FOXO1 | Forkhead box protein O1 | Hs01054576_m1 |
| GAPDH | Glyceraldehyde 3-phosphate dehydrogenase | Hs99999905_m1 |
| GATA3 | Trans-acting T-cell-specific transcription factor GATA-3 | Hs00231122_m1 |
| GZMB | Granzyme B | Hs01554355_m1 |
| GZMH | Granzyme H (cathepsin G-like 2, protein h-CCPX) | Hs00277212_m1 |
| GZMK | Granzyme K (granzyme 3; tryptase II) | Hs00157878_m1 |
| HPRT | Hypoxanthine-guanine phosphoribosyltransferase | Hs02800695_m1 |
| ID2 | DNA-binding protein inhibitor ID-2 | Hs04187239_m1 |
| ID3 | DNA-binding protein inhibitor ID-3 | Hs00954037_g1 |
| IFN-G | Interferon, gamma | Hs00989291_m1 |
| IKZF1 | Ikaros family zinc finger protein 1 | Hs00958474_m1 |
| IL-17A | Interleukin 17A | Hs00174383_m1 |
| IL-4 | Interleukin 4 | Hs00174122_m1 |
| IL13 | Interleukin 13 | Hs00174379_m1 |
| IRF4 | Interferon regulatory factor 4 | Hs01056533_m1 |
| IRF8 | Interferon regulatory factor 8 | Hs00175238_m1 |
| LEF1 | Lymphoid enhancer-binding factor 1 | Hs01547250_m1 |
| NFIL3 | Nuclear factor, interleukin 3 regulated | Hs00993282_m1 |
| PRDM1 | PR domain-containing protein 1 | Hs00153357_m1 |
| PRF1 | Perforin | Hs00169473_m1 |
| RAG1 | Recombination activating gene 1 | Hs01920694_s1 |
| RAG2 | Recombination activating gene 2 | Hs00379177_m1 |
| RORA | RAR-related orphan receptor alpha | Hs00536545_m1 |
| RORC | RAR-related orphan receptor gamma | Hs01076122_m1 |
| RUNX1 | Runt-related transcription factor 1 | Hs00231079_m1 |
| RUNX2 | Runt-related transcription factor 2 | Hs01047973_m1 |
| RUNX3 | Runt-related transcription factor 3 | Hs00231709_m1 |
| SATB1 | Special AT-rich sequence-binding protein 1 | Hs00161515_m1 |
| TBX21 | T-box 21 (TBET) | Hs00894392_m1 |
| TCF3 | Transcription factor 3 (E2A) | Hs00413032_m1 |
| TCF4 | Transcription factor 4 | Hs00162613_m1 |
| TCF7 | Transcription factor 7 | Hs00175273_m1 |
| TCF12 | Transcription factor 12 | Hs00918966_m1 |
| TOX | Thymocyte selection-associated high mobility group box protein TOX | Hs01055573_m1 |
| ZBTB7A | zinc finger- and BTB domain-containing protein 7a (LRF) | Hs00252415_s1 |
| ZBTB7B | zinc finger- and BTB domain-containing protein 7b (THPOK) | Hs00757087_g1 |
| ZBTB16 | Zinc finger and BTB domain-containing protein 16 (PLZF) | Hs00957433_m1 |
| ZNF278 | zinc finger protein 278 (MAZ-related factor) | Hs00204880_m1 |
