# Supplementary material for: CD4+CD8+ T-Lymphocytes in Xenogeneic and Human Graft-versus-Host Disease
Source: Front Immunol. 2020 Nov 24;11:579776. doi: 10.3389/fimmu.2020.579776 (PMC7732609; doi:10.3389/fimmu.2020.579776)
Supplement: Supplementary file 2 [file Table_2.docx]

**Table S2**. Clinical characteristics of the allo-HSCT patients (blood immunophenotyping).

|  | **GVHD (n=24)** | **No GVHD (n=16)** | **p value** |
| --- | --- | --- | --- |
| **Sex** (M:F), no. (%) | 15:9 (62%:38%) | 11:5 (69%:31%) | .35 |
| **Age at SCT**, median (years, range) | 54.4 (16-66) | 41.1 (18-65) | .07 |
| **Type of hematological disease**, no. (%) | | | .2 |
| Acute leukemia | 9 (38%) | 12 (76%) |  |
| MDS | 7 (30%) | 0 (0%) |  |
| MDS/MPN | 2 (8%) | 0 (0%) |  |
| Chronic myeloid leukemia | 1 (4%) | 0 (0%) |  |
| Myelofibrosis | 1 (4%) | 0 (0%) |  |
| Lymphoma | 1 (4%) | 1 (6%) |  |
| Plasma cell disorders | 1 (4%) | 0 (0%) |  |
| Bone marrow failure | 2 (8%) | 2 (12%) |  |
| Others | 0 | 1 (6%) |  |
| **HLA-status of donor**, no. (%) | | | 1 |
| Matched sibling | 24 (100%) | 16 (100%) |  |
| **Stem cell source**, no. (%) | | | .5 |
| PBSC | 17 (71%) | 9 (56%) |  |
| BM | 7 (29%) | 7 (44%) |  |
| **Conditioning regimen**, no. (%) | | | .99 |
| Myeloablative | 8 (33%) | 5 (31%) |  |
| Reduced Intensity | 16 (67%) | 11 (69%) |  |
| **GVHD prophylaxis**, no. (%) |  |  |  |
| Ciclosporine A | 1 (4%) | 5 (31%) | .07 |
| Ciclosporine A + methotrexate | 11 (46%) | 7 (44%) |  |
| Ciclosporine A + mycophenolate mofetil | 11 (46%) | 3 (19%) |  |
| Others | 1 (4%) | 1 (6%) |  |
| **GVHD grading, no. (%)** |  |  |  |
| Grade 1 | 2 (8%) | NA | NA |
| Grade 2 | 17 (71%) | NA |  |
| Grade 3 | 3 (12%) | NA |  |
| Grade 4 | 2 (8%) | NA |  |
