# Supplementary material for: CD4+CD8+ T-Lymphocytes in Xenogeneic and Human Graft-versus-Host Disease
Source: Front Immunol. 2020 Nov 24;11:579776. doi: 10.3389/fimmu.2020.579776 (PMC7732609; doi:10.3389/fimmu.2020.579776)
Supplement: Supplementary file 3 [file Table_3.docx]

**Table S3**. Clinical characteristics of the allo-HSCT patients (rectal and duodenal biopsies).

|  | | **GVHD (n=6)** | **No GVHD (n=6)** | **p value** |
| --- | --- | --- | --- | --- |
| **Sex** (M:F), no. (%) | | 3/3 (50/50) | 2/4 (33/66) |  |
| **Age at SCT**, median (years, range) | | 44 (25-59) | 41 (29-67) |  |
| **Type of hematological disease**, no. (%) | | | |  |
| Acute leukemia | | 1 | 5 |  |
| MDS | | 1 |  |  |
| MDS/MPN | |  |  |  |
| Chronic myeloid leukemia | |  |  |  |
| Myelofibrosis | | 1 | 1 |  |
| Lymphoma | | 2 |  |  |
| Plasma cell disorders | | 1 |  |  |
| Bone marrow failure | |  |  |  |
| Others | |  |  |  |
| **HLA-status of donor**, no. (%) | | | |  |
| Matched sibling | | 2 (33%) | 3(50%) |  |
| **Stem cell source**, no. (%) | | | |  |
| PBSC | | 6 | 6 |  |
| BM | |  |  |  |
| **Conditioning regimen**, no. (%) | | | |  |
| Myeloablative | |  | 4 |  |
| Reduced Intensity | | 6 | 2 |  |
| **GVHD prophylaxis**, no. (%) | |  |  |  |
| Ciclosporine A | |  |  |  |
| Ciclosporine A + methotrexate | | 1 | 4 |  |
| Ciclosporine A + mycophenolate mofetil | | 4 | 2 |  |
| Others | | 1 |  |  |
| **GVHD grading, no. (%)** | |  |  |  |
| Grade 1 | | 1 | NA | NA |
| Grade 2 | | 3 | NA |  |
| Grade 3 | | 2 | NA |  |
| Grade 4 | | 0 | NA |  |
| **Biopsies** | | | |  |
| duodenum | 1 | 5 |  |  |
| sigmoid | 1 | 0 |  |  |
| rectum | 4 | 1 |  |  |
